# Le comportement des médecins généralistes de la ville de Douala au Cameroun face aux troubles dépressifs

**DOI:** 10.11604/pamj.2019.34.37.16715

**Published:** 2019-09-17

**Authors:** Michaël Guy Toguem, Eyoum Christian, Jean-Baptiste Djemo Fotso

**Affiliations:** 1 Université des Montagnes, Bangangté, Cameroun; 2 Hôpital Laquintinie de Douala, Douala, Cameroun

**Keywords:** Capacité diagnostique, dépression, médecin généraliste, obstacles, pratiques, Diagnostic capacity, depression, general practitioner, obstacles, practices

## Abstract

**Introduction:**

Au Cameroun, on constate scepticisme et négligence des médecins à l'égard des patients atteints de troubles mentaux. Pour y apporter une solution, il fallait une appréciation objective. Les troubles dépressifs constituent la forme la plus répandue de troubles mentaux et Douala a le deuxième plus gros effectif de médecins généralistes du pays. Ainsi donc, nous nous sommes proposé d'évaluer les comportements des médecins généralistes de Douala face aux patients atteints de troubles dépressifs.

**Méthodes:**

Nous avons mené une étude descriptive transversale de février à juin 2017 en consultation externe de médecine générale. Pour chaque médecin généraliste inclus, nous faisions remplir à 3 (de ses) patients le «patient health questionnaire version-9» (PHQ-9) pour savoir si le patient était dépressif. Pendant chaque consultation, nous remplissions une fiche de renseignement clinique, pour savoir si le médecin généraliste avait diagnostiqué un trouble dépressif. Si oui, quelle prise en charge il avait adopté. Enfin, nous remettions au médecin généraliste un questionnaire pour connaitre ses difficultés face à la dépression

**Résultats:**

Nous avons obtenu une prévalence de 32,5% des troubles dépressifs en consultation de médecine générale dans la ville de Douala et un taux de diagnostic par les médecins généralistes de 1,92%. Les cas diagnostiqués ont juste reçu des conseils.

**Conclusion:**

Au Cameroun, malgré le faible intérêt accordé aux troubles dépressifs, ils constituent un enjeu de santé publique de par sa fréquence et les effets de morbi-mortalité qu'ils peuvent imposer.

## Abstract (en)

**Introduction:**

In Cameroon, there is medical scepticism and negligence towards patients with mental disorders. Objective assessment might be the solution. Depressive disorders are the most common form of mental disorders and Douala has the second largest group of general practitioners in the country. Thus, the purpose of this study was to evaluate the attitude of general practitioners of Douala towards patients with depressive disorders.

**Method:**

We conducted a cross-sectional descriptive study of general outpatient services from February to June 2017. We asked 3 patients of each general practitioner enrolled in the study to fill the Patient Health Questionnaire version-9 (HQP-9) to assess if they were depressed. During each consultation, we filled a clinical information form to know whether the general practitioner had diagnosed a depressive disorder and, if so, which treatment he had adopted. Finally, we administered to general practitioner a questionnaire to know their challenges of treating depression.

**Results:**

The prevalence of depressive disorders in general practice consultations was estimated to be 32.5% in the city of Douala and the rate of diagnoses made by general practitioners was 1.92%. Diagnosed patients only received some.

**Conclusion:**

Despite the low interest in depressive disorders, in Cameroon they are a public health problem due to their frequency and to depression-related morbi-mortality.

## Introduction

Pendant les années passées dans les hôpitaux, fort a été de constater le scepticisme des médecins et étudiants en médecine à l'égard de la pathologie psychiatrique. Ce qui entraine très souvent une absence de prise en compte, de prise en charge (PEC), une mauvaise PEC, voire un abandon des patients atteints de pathologies psychiatriques. Cette attitude des médecins entraine la dégradation de l'état mental des patients, exclusion, voire décès du patient et le désarroi des proches du patient. Le médecin généraliste constitue la porte d'entrée dans notre système de santé et les troubles dépressifs le groupe de pathologie psychiatrique avec le plus lourd tribu. La dépression est un trouble de l'humeur qui se manifeste par une humeur triste, une perte d'intérêt pour toute activité et une baisse de l'énergie. Elle affecte en permanence 4,4% de la population mondiale; 4,2% des habitants d'Ibadan (Nigéria). La dépression constitue 43,33% des cas de maladie mentale (MM) dans le monde, 10,4% des patients qui consultent en médecine générale dont 15 à 20 % mettent fin à leurs jours [1] et 16,5% en France sur la base du «Patient Health Questionnaire» (PHQ); soient des troubles dépressifs majeurs (TDM) à 9,1%, Trouble dépressif mineur (TDm) à 7,4% et un taux de diagnostic respectif de 75% et de 42,4% [2]. La dépression est classée au 4^ème^ rang des maladies en pourcentage de handicap ajusté aux années de vie et estimés à la 2^ème^position d'ici 2020, pourtant chez des patients suivis par des Médecins généralistes (MGs), les antidépresseurs disponibles sont efficaces, avec un taux de réponse à 6 -8 semaines de 60-70% [3]).

Dans les pays à revenu faible ou intermédiaire 75 à 85% des personnes souffrant de troubles mentaux graves ne reçoivent aucun traitement conventionnel, contre 35 à 59% dans les pays à haut revenu [4]; soit une différence de PEC (Prise En Charge) de 26 à 50%, la PEC de la MM étant majoritairement assurée par des MGs dans les pays développés [5]. Au Cameroun, le ministère de la Santé publique, de commun accord avec le ministère de l'Enseignement supérieur a inclus dans le programme harmonisé national de formation en médecine les enseignements de psychologie médicale, de sémiologie psychiatrique et de pathologie psychiatrique, et FODZO L a diagnostiqué des cas de Troubles dépressifs (TDs) dans la ville de Douala [6]. La ville de Douala, capitale économique du Cameroun, densément peuplée, riche de sa diversité religieuse et ethnique, regroupe des riches et des pauvres. Elle est ouverte au monde par son port et son aéroport international. Elle comptait en 2011, 307 MGs [7] et compte à ce jour 1 psychiatre [8]. Celui-ci est enseignant à la faculté de médecine de la localité et chef du service de psychiatrie de l'Hôpital Laquintinie de Douala; pour une population de 2, 943 millions d'habitants environ en 2015 [9]. Il nous apparait donc évident que nous ne pouvons compter sur le seul psychiatre disponible pour satisfaire cette demande. Donc, il faudrait savoir si la contribution des MGs dans la PEC des patients dépressifs est efficace. A travers des objectifs, une revue de la littérature, et une procédure précise, nous collecterons et discuterons des données afin d'évaluer les comportements des médecins généralistes (MG) de la ville de Douala face aux troubles dépressifs.

## Méthodes

**Lieu de l'étude:** notre étude s'est déroulée dans la ville de Douala, capitale économique du Cameroun, pays de l'Afrique centrale. Elle compte 2 hôpitaux centraux, un hôpital général, 5 hôpitaux de district et plusieurs hôpitaux privés; un Service de Psychiatrie à l'Hôpital Laquintinie de Douala, une faculté de médecine, une faculté de pharmacie et 2 hôpitaux d'application.

**Type d'étude:** nous avons fait une étude descriptive transversale.

**Période d'étude:** notre étude s'est déroulée de février 2017 à juin 2017, soit sur 5 mois

### Population d'étude

***Critères d'inclusion:*** médecins généralistes (MG) en exercice dans la ville de Douala, fiche de consentement éclairé signée; patients: patient en consultation de médecine générale, patient âgé de plus de 11 ans, formulaire de consentement signé.

***Critères d'exclusion:*** médecins généralistes (MG) n'exerçant pas une fonction de clinicien, MG en cycle de spécialisation; patients: patient incapable de communiquer.

***Échantillonnage:*** population d'intérêt: MG exerçant dans la ville de Douala. Le nombre de patients à inclure dans l'étude sera déterminé d'après la formule suivante [10]:

$$n_{p}={\left[\left\{z_{1-\alpha }\sqrt{2\pi (1-\pi )}-Z_{\;\beta }\sqrt{\pi_{1}(1-\pi_{1})+\pi_{2}(1-\pi_{2})}\;\right\}/\Delta \right]}^{2}\text{O}ù\;\Pi =({\text{Π}}_{1}+{\text{Π}}_{2})/2$$

Nous fixons puissance statistique (1-β) requise pour détecter cette différence: 90%. L'application numérique nous donne n = 153, donc le nombre minimal à inclure dans l'étude sera de 153 patients consultant un MG. Le nombre de MGs à inclure dans l'étude sera déterminé selon la formule suivante [11]:

$$n_{mg}=\frac{(\sigma_{1}^{2}+\sigma_{0}^{2}{\left(u+v\right)}^{2}}{{\left(\mu_{1}-\mu 0\right)}^{2}}$$

μ_0_ = 99% et un écart-type σ_0_ = 99%. Nous avons choisi une puissance statistique de 90% et un risque d'erreur α= 5%. Donc, l'application numérique nous donne comme effectif minimal à inclure dans notre étude: 54MGs. Le recrutement a été fait dans les hôpitaux suivants: Hôpital de District de Déido, de Bonassama, de Nylon, de la cité des palmiers, le CMA de Bonamoussadi, de Kotto, à la clinique Saint Padré pio, à la polyclinique de Bonanjo, Daah (centre international d'imagerie médicale et de médecine), le poitier, de l'aéroport, l'Hôpital CEEBEC de Bonabéri, l'Hôpital Catholique Notre-dame de l'Amour, logpom.

### Procédure

À chaque MG voulant faire partie de l'étude, était accordé un délai maximal de 2 jours au cours desquels, nous remettions à 3 de ses patients volontaires une fiche par patient. À la première page figurait le formulaire de consentement éclairé, à la deuxième page le PHQ qui une fois rempli, soit par le patient lui-même, ou en se faisant aider soit par un accompagnant si le patient ne s'exprime qu'en langue, soit par l'enquêteur si des problèmes de compréhension des questions se posait (surtout si le patient est d'expression anglaise). Après cette étape de remplissage, le patient entrait dans le box de consultation. Au cours de cette consultation, nous complétions la troisième page qui porte sur les renseignements cliniques. À la fin, nous soumettions au MG le questionnaire adressé aux MGs.

### Matériel utilisé

#### Questionnaire des médecins généralistes

Il comprend 3 rubriques: 1) profil du MG, qui nous montre les facilitations qui auraient permis au MG d'acquérir les compétences nécessaires en psychiatrie; 2) issues, qui nous permettra de connaitre l'impact de la confrontation entre MG et patient dépressif sur ceux-ci; 3) obstacles, qui nous permettra de connaitre les freins rencontrés lors de la PEC des patients dépressifs.

#### Le patient health questionnaire (PHQ)

Il sera adressé aux patients; c'est un outil Nord-américain d'aide au diagnostic des MMs dont l'usage dans le cadre de la dépression est validé en Europe [12] et reste aussi crédible au Nigéria [13]. Avec une sensibilité de 75% et une spécificité de 90% il permettra d'identifier selon les critères du DSM-IV (Diagnostic and Statistical Manual of Mental Disorders quatrième édition) les patients dépressifs [12].

#### Fiche de renseignements cliniques

Elle servira à déterminer quelles sont les pratiques cliniques des MGs face aux patients dépressifs.

#### Logiciel d'analyse statistique

Les données ont été entrées avec Microsoft Excel 2013 et l'analyse faite avec le logiciel R version 3.3.4

## Résultats

Des 19 établissements hospitaliers de la ville de Douala sollicités, seuls 13 ont bien voulu faire partie de l'étude. Les 13 ainsi inclus sont répartis sur toute la ville de Douala, des quartiers les plus aisés aux plus démunis, incluant une grande diversité: des hôpitaux de districts, des centres médicaux d'arrondissements, des polycliniques, des cliniques de confession protestante, catholique. Dans ces différents hôpitaux, des 56 MGs sollicités, 54 MGs ont bien voulu faire partie de l'étude, soit un taux de participation de 96.43%. Cela nous a permis d'obtenir les différents résultats attendus.

### Fréquence des troubles dépressifs chez les patients qui consultent en médecine générale

Dans la ville de Douala, parmi les patients qui consultent un MG, d'après le PHQ 32.5% sont atteints de TD, dont 10% sont des cas de TDM et 22.5% des cas de TDm.

### Capacité des médecins généralistes à diagnostiquer les troubles dépressifs

Il en ressort que, la probabilité pour un MG exerçant dans la ville de Douala de détecter un TD chez un patient dépressif est de 1,92% (sensibilité). Pour un patient chez qui un diagnostic de TD serait posé par un MG en exercice dans la ville de Douala, la probabilité que ce patient soit effectivement atteint d'un TD est de 33,33% (valeur prédictive positive). D'autres informations relatives à leur capacité diagnostique sont contenues dans le Tableau 1.

**Tableau 1:** Capacité diagnostique des troubles dépressifs par les médecins généralistes de Douala

	Médecins généralistes			
Questionnaire	+	-	Total	
Dépressif	1	51	52	Sensibilité 1,92%
Non dépressif	2	106	108	Spécificité 98,15%
Total	3	157	160	
Valeurs prédictives	Positive 33,33%	Négative 67,52%		

### Pratiques des médecins généralistes face aux troubles dépressifs

Des 160 patients enregistrés, 3 ont été diagnostiqués souffrant d'un TD par les MGs. Parmi ces 3, aucun n'a ni été référé chez le psychiatre, ni reçu une prescription d'antidépresseur ou une psychothérapie. Par contre, ils ont tous reçu des conseils relatifs au diagnostic de dépression, bien que le seul cas de vrai positif souffrait d'un TDM d'après le PHQ.

### Obstacles rencontrés par les médecins généralistes concernant les TDs

#### Profil des médecins généralistes

Parmi les 54 MGs recrutés, 18 (33.3%) ont fait un stage dans un service de psychiatrie. Quarante-cinq sur 53 (84.9%) ont déjà lu sur les TDs, un MG n'ayant pas répondu à cette question; 8 (14,8%) ont assisté à un enseignement post universitaire sur les TDs; 33 (61,1%) ont moins de 5 années, 20 (37%) entre 5 et 15 années, 1 (1,9%) entre 16 et 30 années d'exercice en tant que MG. Aucun d'eux n'exerce ni dans un service de psychiatrie, ni dans un hôpital universitaire. Leurs heures de travail varient entre 8 et 120 heures par semaine avec une moyenne à 47,75 et une médiane à 43.5 heures de travail par semaine. Le nombre de consultation par semaine varie entre 4 et 300, avec une moyenne de 75 et une médiane à 70,68 consultations par semaine. Deux (3,7%) médecins étaient payés à l'acte, 1 (1,9%) à l'heure et 50 (92,6%) par mois sachant qu'il y a eu une abstention de réponse. Les autres caractéristiques sont contenues dans le Tableau 2.

**Tableau 2:** Profil des médecins généralistes de Douala

Variables	Effectifs	Pourcentage
Psychologie Médicale	42	77,8%
Sémiologie Psychiatrique	36	66,7%
Troubles Dépressifs	44	81,5%
Stage en psychiatrie		
≤ 1 semaine	2	3,7%
1-2 semaines	2	3,7%
> 2 semaines	14	25,9%
Pas de réponse	1	1,9%
Lu sur les Troubles Dépressifs	45	83,3%
Enseignement Post Universitaire	8	14,8%
Années exercise		
< 5 ans	33	61,1%
5-15 ans	20	37%
16-30 ans	1	1,9%
Autres services	54	100%
Hôpital non universitaire	54	100%
Mode rémuneration		
Acte	2	3,7%
Heure	1	1,9%
Mois	50	92,6%
Pas de réponse	1	1,9%

#### Issues chez les patients suivis pour troubles dépressifs par les médecins généralistes de Douala

Parmi les 17 MGs qui suivent souvent des patients dépressifs, 3 (18,75%) ont un taux de guérison complète à moins de 25%, 9 (56,25%) entre 25 et 50%, et 4 (25%) supérieure à 50%. Le détail est contenu dans le Tableau 3.

**Tableau 3:** Suivi des patients dépressifs par les médecins généralistes à Douala

Variables	Effectifs	Pourcentage
**Guérison,complète**		
Pas de réponse	38	70,4%
< 25%	3	5,6%
25-50%	9	16,7%
> 50%	4	7,4%
**Guérison,partielle**		
Pas de réponse	37	68,5%
< 25%	9	16,7%
25-50%	3	5,6%
> 50%	5	9,3%
**Satisfaction des soins**		
Pas de réponse	37	68,5%
Non	2	3,7%
Peu	2	3,7%
Assez	11	20,4%
Beaucoup	2	3,7%

#### Obstacles face aux troubles dépressifs

Parmi les obstacles rencontrés par les MGs de la ville de Douala, nous avons un taux de perdu de vue variant entre 0 et 66%, une moyenne de 31,07%, un écart-type de 22,41 et une médiane de 30%. Un MG (1,85%) estime que le psychiatre de la ville n'est pas accessible, mais le reste, 53(98,15%) n'a aucun mal à référer les patients chez qui ils estiment une consultation chez le psychiatre nécessaire. 16 MGs estiment avoir peu, 16 assez, 18 beaucoup et 4 pas du tout besoin de formation sur les TDs d'autres obstacles rencontrés sont contenues dans le Tableau 4.

**Tableau 4:** Obstacles rencontrés par les Médecins Généralistes de Douala face aux patients dépressifs

Variables	Effectifs	Pourcentages
Perdu de vu	mean (sd) = 31.07 (22.41) min < med < max = 0 < 30 < 66	
Besoin de formation		
Non	4	7,4%
Peu	16	29,6%
Assez	16	29,6%
Beaucoup	18	33,3%
Difficulté à referrer	1	1,9%

## Discussion

### Fréquence des troubles dépressifs chez les patients qui consultent en médecine générale dans la ville de Douala

Notre étude a mis en évidence une fréquence des troubles dépressifs en consultation de médecine générale dans la ville de Douala de 32,5%, soit 10% de TDMs et 22,5% de TDms. Il faudrait tout de même noter que notre test a une sensibilité de 75%, une spécificité de 90% et utilise les critères du DSM-IV [12] qui regroupe les TDs en mineur et majeur (DSM 4) contrairement au DSM-V qui est plus détaillé [14]. Nous comparons nos résultats avec ceux d'une étude similaire faite en France avec les mêmes moyens, la même procédure [2] qui avait retrouvé une fréquence 16,5%; soit TDM à 9,1%, TDm à 7,4%. Nous constatons une similitude de fréquence de TDM, ce qui est en accord avec les trouvailles précédentes de l'OMS [1]. Par contre, les TDms sont presque 4 fois plus fréquents à Douala qu'en France, ce qui est contradictoire [1]. Ceci pourrait s'expliquer par plusieurs hypothèses. Les français bénéficient d'une couverture santé pour tous, ce qui motiverait des consultations pour des pathologies débutantes et des symptômes mineurs. Par contre, peu sont ceux qui à Douala ont une assurance maladie, ce qui motiverait des consultations pour des tableaux plus complexes avec par conséquent une plus grande probabilité de regrouper un ensemble de symptômes qui finirait par conduire à une dépression secondaire. Les patients recrutés dans notre étude n'étant pas reçus aux urgences, cela pourrait justifier le fait que leurs symptômes dépressifs n'en soient pas encore au stade majeur pour la plupart. Aussi, la plupart des cas de TDM n'étant pas PEC, ils pourront facilement se chroniciser en des formes mineures ou résiduelles [15], avec une relative accumulation sous cette forme. Donc, ces chiffres élevés seraient dû à un défaut de PEC. Des études ultérieures pourront parmi les cas de TDs, distinguer les TDs primaires des secondaires et évaluer la fréquence des TDs au service des urgences dans la ville de Douala, afin de mieux éclairer notre théorie. Les résultats dans les deux études sont supérieurs à la moyenne mondiale de 10,4% [1], ce qui serait dû à la différence d'outil diagnostique.

### Capacité des médecins généralistes de Douala à diagnostiquer les troubles dépressifs

La sensibilité diagnostique des TDs par les MGs de la ville de Douala est de 1,92%, quand les MGs de France sont à 75% pour les TDMs et de 42% pour les TDms [2], il y a de quoi se poser des questions. Il faudrait quand même reconnaitre que la présence d'une troisième personne pendant la consultation pourrait limiter la mention par le patient de symptômes psychiques, ce qui sous estimerait cette valeur. La différence dans les deux études pourrait être justifiée par le fait que la plupart des MGs disaient ne pas du tout s'intéresser aux pathologies psychiatriques lors de leurs consultations. Ils reconnaissaient que ces pathologies passeraient ainsi inaperçues, à moins d'être flagrantes, auquel cas ils réfèreraient directement chez le psychiatre. Le manque d'intérêt à leurs avis étant dû au fait que la psychiatrie leur paraît comme un mystère. Ce qui soulève des questions de formation de ces MGs par rapport à la dépression. Le seul cas de vrai positif pourrait avoir été diagnostiqué par ce que celui-ci souffrait d'un TDM, donc avait un tableau plus expressif, bien que la PEC n'est pas été correcte ou être le seul fruit du hasard. Donc, très peu de MG à Douala réussissent à diagnostiquer les TDs par ce qu'ils ne s'y intéressent pas assez et manquent de savoir sur les TDs. Nous reviendrons sur les éventuelles causes de cette situation plus bas.

### Pratiques des médecins généralistes face aux troubles dépressifs

Des 3 cas diagnostiqués dépressifs par 3 MGs, aucun n'a été référé chez le psychiatre et ont juste reçu des conseils. Ceci ne constitue qu'un petit aperçu, compte tenu du faible effectif des MGs ici considéré. Cette attitude pourrait s'expliquer soit par le fait qu'ils auraient un doute sur le diagnostic, soit parce qu'ils espéraient que cela puisse finir spontanément, avec les conseils ou avec la guérison de la pathologie organique. De ces trois cas, 2 ont reçu un rendez-vous, mais, il reste encore à savoir si cela est lié seulement à la pathologie organique pour laquelle ces patients étaient suivis ou si cela tenait aussi compte du diagnostic de TD posé. Mais, compte tenu des propos des MGs qui disaient référer le plutôt possible, on pourrait se dire qu'ils auraient pour habitude de référer les cas dont ils sont convaincus d'une atteinte psychiatrique, ceci quand le tableau est flagrant. Donc, il est plus probable que ces rendez-vous n'aient tenu compte que de la pathologie organique, ce qui est bien en accord avec les études précédentes. Ainsi, les MGs de Douala ne prendraient en charge leurs patients dépressifs que par des conseils et des références mais, d'amples études devront être faites pour avoir des réponses plus fiables.

### Obstacles rencontrés par les médecins généralistes concernant les troubles dépressifs

Le seul MG qui a eu un vrai positif, et 2 vrais négatifs, donc tous les 3 diagnostics étaient en accord avec le PHQ, a reçu une formation complète comprenant : psychologie médicale, sémiologie psychiatrique, TDs et un stage en psychiatrie. Il a lu sur les TDs, n'a jamais assisté à une formation post universitaire sur les TDs, exerce depuis 5 à 15 ans et est en exercice dans un hôpital non universitaire, dans un service autre que le service de psychiatrie. Il travaille 30 heures par semaine, avec une moyenne de 87,5 consultations par semaine soit un rapport de 2,92 consultations par heure ce qui est largement au-dessus de la moyenne qui est de 1,48 consultations par heure. Ce MG est rémunéré à l'acte contrairement à la grande majorité et a un taux de guérison complète compris entre 25 et 50%, et partiel inférieur à 25%. Il est assez satisfait des soins qu'il prodigue à ses patients dépressifs, avec un taux de perdu de vue de 40%, ce qui est bien au-dessus de la moyenne de 31,07%, bien que cette moyenne ne soit pas du tout fiable, compte tenu du taux de faux positifs. Peut-être qu'il les perdrait de vue après leur avoir proposé une référence chez le psychiatre, comme il dit le faire très souvent, chose que les patients dépressifs ont très souvent du mal à accepter. Ce MG estime tout de même avoir assez besoin d'une formation dans PEC des TDs, ce qui pourrait aussi signifier une disposition à apprendre. Les informations précédentes ayant été recueillies sur des questionnaires, il existe un biais de mémoire et il serait difficile de tirer des conclusions fiables à partir du profil d'un seul MG. Des 30 autres MGs ayant consulté au moins un patient dépressif d'apprêt le PHQ, dont 9 ayant reçus une formation complète; aucun n'a diagnostiqué un TD chez ces patients. Ce qui laisse penser que le contenu des formations devant préparer les MGs à diagnostiquer et PEC les TDs serait inadapté, sans compter que 43 (79,63%) MGs ont reçu une formation incomplète sur les TDs.

## Conclusion

Nos résultats soulignent la fréquence élevée des TDMs, soit 10% en consultation de médecine générale dans la ville de Douala tout comme dans la plupart des pays [1]. On note une fréquence nettement plus élevée des TDms en consultation de médecine générale dans la ville Douala, soit 22,5%. Malgré la très faible capacité diagnostique des TDs par les MGs de Douala, soit une sensibilité de 1,92%. Les conseils et la référence constituent les PECs les plus utilisées. Ces données sont en accord avec les autres pays africains [16]. Cette incapacité à faire face aux TDs entraine une souffrance croissante des populations et remettent en question la qualité du contenu des formations des MGs sur les TDs en particulier et sur les pathologies mentales en général au Cameroun.

### Etat des connaissances actuelles sur le sujet

- L'existence des troubles dépressifs à Douala.

### Contribution de notre étude à la connaissance

- Fréquence des troubles dépressifs en consultation de médecine générale dans la ville de Douala;
- Taux de diagnostic des troubles dépressifs par les médecins généralistes de Douala en consultation externe.

## Conflits d’intérêts

Les auteurs ne déclarent aucun conflit d'intérêts.

## Remerciements

Au Dr SIMO Josué, pour l'analyse statistique, au délégué régional de la santé du littoral Cameroun, qui a bien voulu que cette étude soit faite sur l'étendue de son territoire, aux directeurs des différents hôpitaux qui ont bien voulu faire partie de cette étude, aux médecins généralistes et patients de la ville de Douala pour leur bonne volonté et leur hospitalité.
